# Effects of Ceftiofur and Chlortetracycline on the Resistomes of Feedlot Cattle

**DOI:** 10.1128/AEM.00610-18

**Published:** 2018-06-18

**Authors:** Margaret D. Weinroth, H. Morgan Scott, Bo Norby, Guy H. Loneragan, Noelle R. Noyes, Pablo Rovira, Enrique Doster, Xiang Yang, Dale R. Woerner, Paul S. Morley, Keith E. Belk

**Affiliations:** a Department of Animal Sciences, Colorado State University, Fort Collins, Colorado, USA; b Department of Veterinary Pathobiology, Texas A&M University, College Station, Texas, USA; c Department of Large Animal Clinical Sciences, Michigan State University, East Lansing, Michigan, USA; d Department of Animal and Food Sciences, Texas Tech University, Lubbock, Texas, USA; e Department of Clinical Sciences, Colorado State University, Fort Collins, Colorado, USA; f Instituto Nacional de Investigacion Agropecuaria, Treinta y Tres, Uruguay; g Department of Animal Sciences, University of California—Davis, Davis, California, USA; INRS—Institut Armand-Frappier

**Keywords:** antibiotic resistance, antimicrobial agents, cattle, feedlot, metagenomics, postantibiotic effect

## Abstract

Treatment of food-producing animals with antimicrobial drugs (AMD) is controversial because of concerns regarding promotion of antimicrobial resistance (AMR). To investigate this concern, resistance genes in metagenomic bovine fecal samples during a clinical trial were analyzed to assess the impacts of treatment on beef feedlot cattle resistomes. Four groups of cattle were exposed, using a 2-by-2 factorial design, to different regimens of antimicrobial treatment. Injections of ceftiofur crystalline-free acid (a third-generation cephalosporin) were used to treat all cattle in treatment pens or only a single animal, and either chlortetracycline was included in the feed of all cattle in a pen or the feed was untreated. On days 0 and 26, respectively, pre- and posttrial fecal samples were collected, and resistance genes were characterized using shotgun metagenomics. Treatment with ceftiofur was not associated with changes to β-lactam resistance genes. However, cattle fed chlortetracycline had a significant increase in relative abundance of tetracycline resistance genes. There was also an increase of an AMR class not administered during the study, which is a possible indicator of coselection of resistance genes. Samples analyzed in this study had previously been evaluated by culture characterization (Escherichia coli and Salmonella) and quantitative PCR (qPCR) of metagenomic fecal DNA, which allowed comparison of results with this study. In the majority of samples, genes that were selectively enriched through culture and qPCR were not identified through shotgun metagenomic sequencing in this study, suggesting that changes previously documented did not reflect changes affecting the majority of bacterial genetic elements found in the predominant fecal resistome.

**IMPORTANCE** Despite significant concerns about public health implications of AMR in relation to use of AMD in food animals, there are many unknowns about the long- and short-term impact of common uses of AMD for treatment, control, and prevention of disease. Additionally, questions commonly arise regarding how to best measure and quantify AMR genes in relation to public health risks and how to determine which genes are most important. These data provide an introductory view of the utility of using shotgun metagenomic sequencing data as an outcome for clinical trials evaluating the impact of using AMD in food animals.

## INTRODUCTION

Globally, antimicrobial-resistant bacteria have been recognized as a concern (1). As a result, various uses of antimicrobial drugs (AMD) in agriculture have received increasing scrutiny and criticism (2–4). Antimicrobial drugs are used in animal agriculture for treatment, control, and prevention of disease, as well as to improve efficiency of food production (5). Concerns about the use of AMD in food-producing animals revolve around the fear that antimicrobial-resistant bacteria, along with their resistance elements, present in beef cattle could negatively affect public and environmental health.

Antimicrobial drugs are used in beef cattle for treatment, control, and prevention of bacterial infections, which can directly result in animal health and welfare benefits and can also indirectly improve production efficiency. Two commonly used drugs in cattle in the United States are ceftiofur (a third-generation cephalosporin), which is administered parenterally, and chlortetracycline (CTC), which is administered by inclusion in feed. Previously, a research group found that CTC treatment in feedlot cattle without prior ceftiofur (administered as ceftiofur crystalline-free acid [CCFA]) exposure resulted in a decreased likelihood of recovering ceftiofur-resistant Escherichia coli isolates (6). As such, that research group hypothesized that CTC might antagonize proliferation of cephalosporin-resistant enteric bacteria in ceftiofur-treated cattle. The effects of combined CCFA and CTC treatment were therefore investigated in beef feedlot cattle enrolled in a clinical trial based on a 2-by-2 factorial treatment design (7, 8). These studies used cultured E. coli isolates to evaluate *bla*_CMY-2_, *tet*(A), and *tet*(B) and quantitative PCR (qPCR) in metagenomic fecal DNA extractions to identify and quantify the presence of the aforementioned antimicrobial resistance (AMR) genes in addition to *bla*_CTX-M-32_. Their results suggested that both treatments were associated with both increased prevalence and quantity of ceftiofur resistance.

Research conducted using clinical trials to evaluate the impacts of AMD administration provides better opportunities to avoid bias, and these studies conducted by Kanwar et al. (7, 8) and Ohta et al. (9) provide important information that is relevant to beef production and public health. However, they provide limited information about the entire microbiome and resistome, given their focus on either specific bacterial species (e.g., E. coli and Salmonella) or a few AMR genes [e.g., *tet*(A), *tet*(B), *bla*_CMY-2_, and *bla*_CTX-M-32_] using qPCR. Since this field work was completed, advances in the application of shotgun sequencing and bioinformatics for characterization of the microbial metagenome have made it possible to investigate AMR ecology in the context of the predominant microbiome and resistome. While the four genes previously studied by Kanwar et al. (7, 8) are important, there are thousands of additional genetic determinants of AMR that might be found in the microbial milieu of the gut. Shotgun metagenomic sequencing provides an opportunity to investigate dynamics in the ecology of AMR through a much broader investigative lens.

The objective of this study was to use shotgun metagenomic sequencing to investigate the changes in the fecal resistome of feedlot cattle using samples collected during a previously conducted clinical trial that administered CCFA and CTC in feedlot cattle (7, 8). A secondary objective of this study was to compare conclusions based on shotgun metagenomics to qPCR and culture results from the same samples.

## RESULTS AND DISCUSSION

### General sequencing results.

Sequencing of 32 composite DNA samples extracted from feces of cattle generated 1.42 billion total reads with an average of 44.24 million reads (average read length, 126 bp) per sample (range, 14.62 to 67.87 million reads). Phred scores across all samples averaged 35.11 (range, 32.6 to 35.77). Trimming resulted in removal of 2.4% of reads across all samples. Of the remaining trimmed reads, 0.14% were classified as Bos taurus or *Bos indicus* and removed. Sequencing depth was considered appropriate through the construction of rarefaction curves for both the species and AMR genes present (see Fig. S1 in the supplemental material).

### Overall resistome composition.

**FIG 1:**
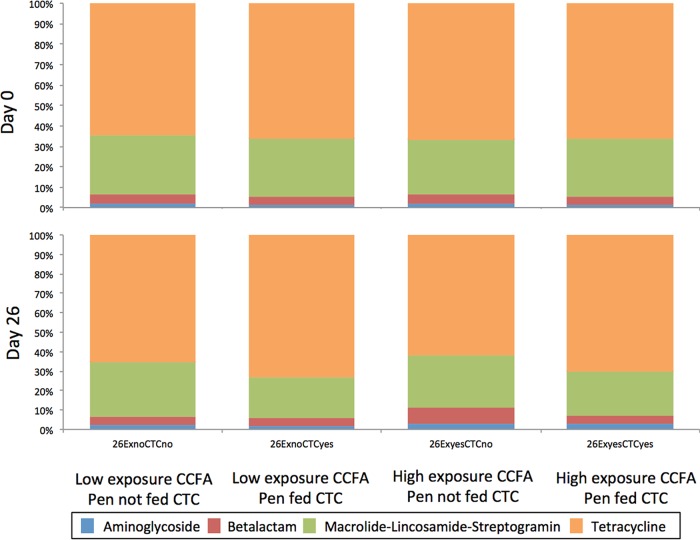
Normalized relative abundances of classes of antimicrobial resistance (combined across treatment day and treatment) treated in a 2-by-2 factorial of chlortetracycline (yes or no) or ceftiofur crystalline-free acid (low exposure, with one animal treated in the pen, or high exposure, with all animals treated in a pen). Each column represents a treatment on day 0 and day 26.

A total of 1.25 million reads aligned to 101 AMR genes included in the MEGARes database (10). The AMR-aligned reads accounted for 0.09% of total mapped reads though this total was affected by antibiotic treatment (Table 1). Sequences identified in these samples were classified hierarchically as hits to AMR genes (i.e., at the drug class, mechanism, and group levels), with hits assigned to five unique classes of resistance (aminoglycoside, β-lactams, macrolides-lincosamides-streptogramins [MLS], tetracyclines, and mechanisms for multiple drugs [e.g., efflux pumps]), in which there were genes encoding 13 different mechanisms (Table S1). Hits aligning to tetracycline resistance genes were the most common among normalized reads mapping to the differing AMR gene classes (8.19 tetracycline resistance hits/12.64 total AMR gene-normalized counts as a ratio to bacterial 16S gene-normalized counts), while MLS genes comprised most of the remaining AMR genes (3.30 MLS resistance hits/12.64 total AMR gene-normalized counts as a ratio to bacterial 16S gene-normalized counts) (Fig. 1). The principal genes conferring mechanisms of resistance to the tetracycline class (98.5%; 8.07/8.19 tetracycline AMR gene-normalized counts as a ratio to bacterial 16S gene-normalized counts) were for ribosomal protection proteins (RPP), with the most abundant tetracycline groups being *tet*(Q) (39.5% of the resistome) followed by *tet*(40) (5.3% of the total resistome). Macrolide resistance efflux pump (MREP) genes were the main mechanism that conferred resistance in the MLS class (88.7%; 2.93 MREP-resistant bacteria/3.30 MLS AMR gene-normalized counts as a ratio to bacterial 16S gene-normalized counts). The other commonly encountered mechanism of resistance was to Ambler class A β-lactamases, which comprised 4.6% of total resistance mechanisms (primarily made up of *bla*_CFX-A6_ and the *bla*_ACI-1_ groups). Furthermore, resistance genes that Noyes et al. (11) defined as important to human health care were not found in these samples.

**TABLE 1:** Percentage of raw sequence reads in relation to raw hits to antimicrobial resistance genes by day and chlortetracycline treatment^*a*^

Treatment day and CTC use^*b*^	Estimated percentage of aligned reads^*c*^
0	
No	0.077 A
Yes	0.077 A
26	
No	0.080 A
Yes	0.115 B
Mean square error	0.004

a Ceftiofur crystalline free-acid (CCFA) effects were also considered as part of an interaction and as a main effect and was found to not have a significant impact on the resistome. Thus, estimates reported here are not separated by CCFA treatment.

b CTC, chlortetracycline.

c Values are least square means. Values with different letters are significantly different (*P* < 0.05).

Interestingly, tetracycline resistance also predominated among phenotypically characterized E. coli recovered during prior investigations of these same samples (7); phenotypic resistance to tetracycline was identified in 61.1% of isolates. Other metagenomic sequencing studies have also identified tetracycline to be the predominant class of resistance genes found in the fecal microbiome of cattle, followed by resistance to the MLS drug class (11, 12); since resistance to the latter class is uncommon in Gram-negative bacteria, there is no prior point of comparison in this study.

### Treatment with tetracycline was associated with tetracycline AMR genes.

**FIG 2:**
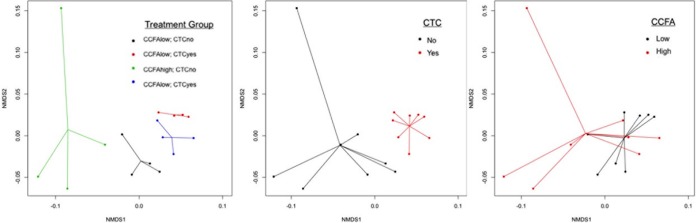
Nonmetric multidimensional scaling (NMDS) ordination plots of resistome composition on day 26 by treatment group (stress = 0.096, *R* = 0.58, and *P* = 0.001), chlortetracycline (CTC) treatment (stress = 0.082, *R* = 0.49, and *P* = 0.001), and ceftiofur crystalline-free acid (CCFA) treatment (stress = 0.084, *R* = 0.12, and *P* = 0.067). Significance of CTC treatment and no CCFA treatment seems to show that CTC treatment was the main driver of treatment group resistome differences.

**FIG 3:**
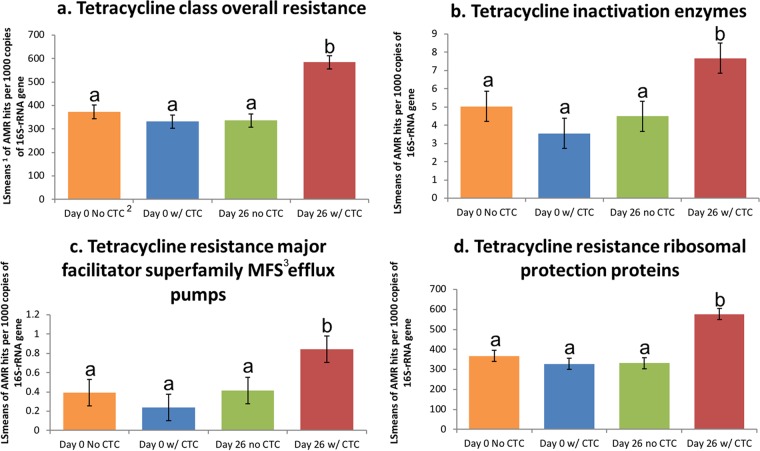
Normalized relative abundance counts per 1,000 copies of the bacterial 16S gene by (a) tetracycline resistance overall as a class and the three mechanisms of tetracycline resistance, (b) tetracycline inactivation enzymes, (c) tetracycline resistance major facilitator superfamily (MFS) efflux pumps, and (d) tetracycline resistance ribosomal protection proteins displayed over day 0 with no animals fed CTC, day 0 with (w/) all animals in the pen fed CTC, day 26 with no animals fed CTC, and day 26 with all animals in the pen fed CTC. The ratio estimates how many tetracycline resistance genes are present per 1,000 bacteria. Within the panels, letters above the bars that differ denote a significant (Bonferroni adjusted *P* value, <0.05) difference. ^1^, LSMeans, least square means; ^2^, CTC, chlortetracycline; ^3^, MFS, major facilitator superfamily.

The resistome increased in size as a result of CTC treatment (Table 1), with a significantly increased number of normalized hits to AMR genes in the fecal resistome of the two groups of cattle treated with CTC (*P* < 0.05) compared to the number of hits in those not treated with CTC. Additionally, the relative abundance of tetracycline resistance genes increased (Bonferroni adjusted *P* value, <0.05) from day 0 to day 26 among groups of cattle fed CTC regardless of CCFA treatment (Fig. 1 and 2). The increase in tetracycline resistance genes was significant among all mechanisms of tetracycline resistance that were identified (Fig. 3). Noyes et al. (11) also found an increase in the proportion of samples positive for two mechanisms of tetracycline resistance (major facilitator superfamily efflux pumps and ribosomal protection proteins) in feedlot pens where at least one animal was administered tetracycline during feeding. While this study and that of Noyes et al. (11) would suggest that tetracycline class administration exerts selective pressure on tetracycline-resistant bacteria, the existing literature is not as clear on the topic. For example, Morley et al. (13) found phenotypes of resistant non-type-specific E. coli cultured from feces of beef feedlot cattle increased in tetracycline resistance prevalence as the feeding period progressed despite the direct lack of exposure to tetracycline. A possible driver of this increase could have been the historic tetracycline use in feedlots, resulting in elevated environmental levels prior to the study. These various associations between resistance and exposure to tetracycline limit the ability to form strong conclusions about the influence it has on promotion of AMR in cattle. Another consideration when changes to tetracycline resistance are evaluated is the time from exposure to sampling. Samples evaluated in this study were collected on day 0 and day 26, which was only 6 days after the final in-feed administration of CTC. As such, these results may represent a maximal impact of exposure in comparison to situations when there is a longer washout period.

### β-Lactam resistance saw no long-term changes in resistance patterns.

While normalized counts of hits to β-lactam resistance genes were slightly higher on day 26 in study groups where all cattle were treated parenterally with CCFA, these differences were small and not statistically significant (Bonferroni adjusted *P* value, ≥0.05) (Fig. 1 and 2). At the mechanism level, on day 26, there were no significant differences between treatment groups in either Ambler class A or Ambler class C (Bonferroni adjusted *P* value, ≥0.05). At the group level, CMY and CTX were not found in the data; thus, no formal statistical analysis could be conducted. The CFX group was the predominant group of β-lactam resistance, and there was no significant difference between treatment groups on day 26 (Bonferroni adjusted *P* value, ≥0.05). These findings are in contrast to the findings of studies using culture to study *bla*_CMY-2_ (Ambler class C) and qPCR to study both *bla*_CMY-2_ and *bla*_CTX-M_ (Ambler class A) genes in the same samples (7, 8). This difference may be related to the shotgun sequencing approach used here, which encompassed the investigation of over 2,000 β-lactam gene targets in the database used. Using metagenomic sequencing, we found that hits to *bla*_ACI-1_ and *bla*_CFX-A6_ (both Ambler class A) comprised the majority of hits to β-lactam resistance genes, representing 32.4% and 52.7% of the total β-lactams found, respectively.

The differences in results regarding β-lactam resistance genes identified in the same samples as reported by Kanwar et al. (7, 8) and the results reported here highlight a currently unanswered question in AMR research: which resistance genes are most impactful for human health? At the molecular class level, β-lactam genes are classified in subgroups A through D (14). The *bla*_CMY-2_ gene belongs to subgroup C, and *bla*_CTX-M-32_ belongs to subgroup A. Both of these genes are considered highly important when found in pathogens because of their ability to confer resistance to higher-order β-lactams such as third- and fourth-generation cephalosporins, which are important for treating infections resistant to lower-order β-lactams, such as penicillin, aminopenicillins, and first- and second-generation cephalosporins (15). In contrast, *bla*_ACI-1_ and *bla*_CFX-A6_ are both class A β-lactam resistance genes that encode resistance that is greater against lower-order β-lactams and have mainly been described in Gram-positive anaerobes (16). Higher-order β-lactam drugs have been classified by the World Health Organization and other public health groups as being “critically important” to human medicine, while lower-order β-lactam drugs have a lower classification as “highly important” (17). While these designations relate to the availability of alternatives for treating resistant infections, they do not account for the probability of encountering bacteria with resistance to these drugs. This raises the question of whether it is riskier to have more nonpathogenic bacteria resistant to a highly important antimicrobial or fewer pathogenic bacteria resistant to highest-priority, critically important antimicrobials. To this end, it is unclear if the researcher should focus solely on the most prevalent or highly abundant resistance genes or on those that confer resistance to the drugs of last resort.

### Relative abundances of resistance genes increase for AMD not administered to the population.

Three other classes of resistance were present at a high enough level to compare formally between treatment days. The relative abundances of AMR genes encoding aminoglycoside resistance increased (Bonferroni adjusted *P* value, <0.05) from day 0 to day 26 in all treatment groups (Table 2). Over this same time period, the MLS class and multidrug resistance did not see a significant increase (Bonferroni adjusted *P* value, >0.05). An increase in aminoglycoside resistance is of interest because no aminoglycosides were administered to the cattle throughout the study. Noyes et al. (11) also found aminoglycoside resistance changed in their study absent treatment with this AMD. These results suggest that other ecological factors drove this change, such as changes in the microbiome or coselection, similar to the change described by Coque et al. (18) regarding the proliferation of extended-spectrum β-lactamase (ESBL) resistance through coselection with aminoglycosides. In this instance, the most common hits to AMR genes for aminoglycoside drugs encoded mechanisms for drug modification via O-phosphorylation.

**TABLE 2:** Least square means of antibiotic classes of resistance not administered in the study

Class of resistance^*a*^	Relative abundance (least square means) by treatment day^*b*^		SEM
	0	26	
Aminoglycoside	9.5 A	17.0 B	1.4
MLS	139.8 A	170.4 A	14.1
MDR	0.9 A	0.4 A	0.5

a While other classes of resistance were identified, they were not present at a high enough level to be formally statistically compared. MLS, macrolide-lincosamide-streptogramin B; MDR, multidrug resistance.

b Values were determined per 1,000 copies of the 16S rRNA gene on day 0 and day 26 pooled across all treatment combinations. Means within rows with different letters are significantly different (Bonferroni adjusted *P*, <0.05).

### Treatments had little impact on resistome richness, but CCFA administration was associated with changes in diversity.

Among the four treatment groups, there were no significant differences detected in richness of the resistome at the class, mechanism, or group levels (*P* = 0.31, *P* = 0.52, and *P* = 0.11) though due to the pooling of samples for shotgun metagenomic evaluation, this outcome may have been different with a larger sample size. At the class level in pens not treated with CTC, pens of cattle where only 1 animal was treated with CCFA had higher (*P* < 0.05) resistome diversity than pens of cattle where all cattle were treated with CCFA.

While changes in microbiome diversity as a result of antibiotic administration have been well documented in both human and animal models, there has been less research on the impact of administration on the resistance genes in the community. Zhu et al. (19) found that the use of antibiotics on swine farms increased the diversity of AMR genes found. Here, it was found that in pens of cattle where only one animal was treated with CCFA (as opposed to all the cattle in a pen), there was a higher diversity of AMR genes.

These differences in diversity could be due to relative fitness costs. Sun et al. (20) hypothesized that resistance occurs at a relative fitness cost. Commensal bacteria external to the animal and that are susceptible to AMD may outcompete within-host resistant bacteria after AMD treatment ends or mitigate an expansion of *ex vivo* resistant bacterial populations immediately posttreatment. While this was not observed in previously described culture studies (7–9), this repopulation is likely dependent on the availability of bacteria from the pen environment. In this study, higher group-level exposures to CCFA (an extended-duration formulation of ceftiofur) may have had a greater long-term suppressive effect on enteric bacteria and, thus, a lower measure of population diversity.

### The potential for bias associated with sequence classification.

Investigations on whether methods used for alignment and classification of sequencing reads could have biased the study conclusions, especially given that the treatments used in the clinical trial were a cephalosporin (a β-lactam) and a tetracycline, were also conducted. In order to evaluate the relative abundance of AMR genes in fecal samples, our bioinformatics pipeline used a Burrows-Wheeler alignment (BWA) tool, as previously described (10, 21). There are a greater number of published β-lactam resistance genes with a high degree of sequence homology than the published sequences for tetracycline class resistance genes, and this difference in numbers of published genes combined with differences in the likelihood of genes having conserved sequences creates the potential to differentially influence estimates of relative abundance and diversity for these two classes of antibiotic resistance.

While it is important to use a comprehensive database of reference sequences for resistance genes in order to fully describe the diversity of features within the resistome, the presence of sequence homology across different features can lead to artificial inflation of the number and diversity of features to which hits are attributed. If the sequence of a read can be equally attributed to multiple features, alignment tools such as BWA will typically randomly assign the read to one of the potential classifications, thereby tending to increase diversity. Unfortunately, the potential for this to affect classification of hits to AMR genes differs among classes of AMD. Accepted nomenclature rules for identifying new tetracycline AMR genes suggest that a newly identified gene must have ≤80% amino acid identity with a previously described gene (22). In contrast, accepted nomenclature rules for β-lactam AMR genes suggest that the predicted protein sequence for new genes may differ by only one or more amino acids from previously described genetic determinants (23). This is illustrated by TEM-20, which differs from TEM-19 by a single silent mutation while TEM-21 differs from TEM-3 and TEM-14 by a single mutation (24). Other classes of AMR genes do not have uniformly accepted nomenclature guidelines. Decreasing the number of substitutions that are required to name a new gene can therefore lead to a publication bias for AMR genetic elements, as reflected in different AMR gene databases. For example, MEGARes includes 2,138 AMR gene accession numbers associated with β-lactam resistance (55.9% of the entire MEGARes database) but only 143 AMR gene accession numbers associated with tetracycline resistance (3.7% of the database).

This bias was investigated by running a second analysis in which a read was allowed to be classified against multiple AMR database elements if it aligned equally well to a homologous sequence contained in multiple accession numbers (thus a single read had the potential to contribute a hit to multiple gene accession numbers). The focus of this analysis was on tetracycline and β-lactam resistance. The objective of this sensitivity analysis was to determine if small shifts in β-lactam and tetracycline resistance gene relative abundances were masked by our bioinformatics approach. However, analysis of this revised data set did not reveal any different outcomes from our original analysis.

### Comparisons to culture and PCR results.

After the first alignment to the AMR database, the most commonly identified resistance elements identified using metagenomic sequencing differed from the genes targeted by qPCR analysis used in the previous investigation of these fecal samples (7, 8). To verify the absence of these genes, all 32 sample sequences were visually evaluated for primers specific to the previously studied genes. The previous studies reported shifts in abundances relative to *tet*(A), *tet*(B), *bla*_CMY-2_, and *bla*_CTX-M_ (qPCR only), genes also identified by qPCR in E. coli isolates and in metagenomic DNA extractions. However, shotgun sequencing found no reads that aligned to the *tet*(B), *bla*_CTX-M_, or *bla*_CMY-2_ gene, and only one sample exhibited a hit to the *tet*(A) gene. This finding was confirmed by visual inspection of sequences (see Fig. S2 in the supplemental material).

The inability to identify sequences in shotgun sequencing data that were found using qPCR is not unexpected and highlights differences in research questions that may be better approached using these two tools. A fundamental feature of PCR testing is the amplification of specific nucleotide sequences, which can be further enhanced if culture methods are used to select or enrich different parts of the microbiome. In contrast, a foundational assumption of shotgun sequencing is that the sequence abundances that are generated in the analysis are directly proportional to their relative abundances in the original sample. While a reflective sample of the community, shotgun metagenomics typically highlight the predominant bacteria and genes present, with rare genes less likely to be sequenced. Thus, if the sequences targeted in selected PCR assays are relatively rare in the metagenome, they can easily remain undetected by shotgun sequencing. Therefore, while the breadth of metagenomics allows the measurement of the most prevalent resistance genes in a bacterial community, it is in no way a tool able to characterize every resistance gene in a sample. This leads to a fundamental question of whether AMR genetic elements are important in addressing the research question even when they are extremely rare. If common background elements can be disregarded and if rare AMR genes are more important to public health than these common elements, then PCR may be a better tool for interrogating AMR gene ecology than shotgun sequencing. Comparison of these approaches raises another important question, which is whether we know enough about the ecology of AMR to focus on a few genes using PCR as opposed to characterizing all possible AMR genes using shotgun sequencing. In any complex bacterial community, there are many different types of bacteria and different AMR genes within the microbiome, as indicated by previous research (11, 12, 25). Shotgun metagenomics allows the simultaneous study of pathogenic and commensal bacteria; importantly, the latter dominate in the enteric ecology of all but a very few clinical diseases. Yang et al. (25) demonstrated that while shotgun metagenomics is not yet appropriate for regulatory supervision of pathogens, it is an appropriate tool for pathogen screening. The bacterial community component of metagenomics is especially important as horizontal gene transfer of attributes to increase cell survival, such as antibiotic resistance, has been well established (26), with some commensal bacteria serving as a reservoir for these genes (27). As a result, characterization of community antibiotic resistance should be performed within the broader bacterial community, ideally not be limited to a single indicator organism, and preferably not be limited to a single methodological approach.

### Conclusion.

Shotgun metagenomic sequencing provided an ecological perspective on the microbial dynamics related to AMR in this clinical trial comparing two different AMD treatments in beef cattle. There were no significant detectable changes in the relative abundances of β-lactam resistance genes in the feces of cattle in association with treatment with CCFA although diversity was impacted. In contrast, the relative abundances of tetracycline resistance genes increased on day 26 after exposure of the cattle to chlortetracycline via feed. There was also a detectable increase in the relative abundances of aminoglycoside-resistant genes at the end of the trial in all cattle. However, these results did not mimic those obtained when feces were evaluated using culture and PCR (7, 8). These various results highlight the complex nature of community antibiotic resistance that cannot be attributed solely to antibiotic selective pressure. Shotgun metagenomics provided a robust characterization of resistance genes present in the enteric bacterial community and provided a contrasting view of the dynamics of antimicrobial resistance to that provided via either indicator or pathogenic bacterial culture or else more targeted PCR amplifications.

## MATERIALS AND METHODS

### Study design.

Collection of samples used in this study has been previously described (7, 8). Briefly, bovine fecal samples used in this study were collected per rectum on two sampling days over a 26-day feeding period. Two replicates each consisting of 88 steers were blocked by weight and randomly assigned to eight pens that each housed 11 steers. The steers were predominantly of the Angus breed and were yearlings with an average weight of 437.3 kg at the beginning of the study. All steers were housed in dry-lot pens and fed a flaked-corn-based diet with added roughage, protein, vitamins, and minerals. Thus, the study consisted of a total of 176 cattle. In each replicate, pens of cattle were randomly assigned to one of four treatment groups, based on a 2-by-2 factorial design. One treatment group received chlortetracycline via the feed (Aureomycin; Alpharma, Bridgewater, NJ, USA) at a level intended to deliver 22 mg/kg of body weight of chlortetracycline per day for 23 days (chlortetracycline was administered during three separate 5-day periods, with a 1-day break in between feeding periods, starting on day 4 with the final treatment on day 20), and all cattle in these pens were treated parenterally per the manufacturer's instructions on day 0 with long-acting CCFA (Excede; Zoetis Animal Health, NJ, USA) at a dose of 6.6 mg/kg body weight. All cattle in the second treatment group received chlortetracycline in feed, but only 1 of the 11 steers in each pen was treated on day 0 with CCFA. The third treatment group was not treated with chlortetracycline, but all cattle were treated parenterally on day 0 with CCFA. The fourth treatment group was also not treated with chlortetracycline, and only 1 of the 11 steers in each pen was treated on day 0 with CCFA.

### Sample collection and processing.

Only the feces collected per rectum on day 0 and day 26 from all animals were included in this analysis. DNA was extracted from 200 mg of feces from each sample using a QIAamp DNA stool minikit (Qiagen, Valencia, CA, USA) according to the manufacturer's instructions using a QIAcube robot (Qiagen, Valencia, CA, USA). Once DNA was extracted, an equal mass of DNA from each sample was pooled from each animal by pen and day; thus, a total of 32 composite samples were prepared (the DNA used in the metagenomic analysis was from the same extraction as the culture and qPCR results). Quality and concentration of the DNA were determined using a NanoDrop ND-1000 UV-visible light spectrophotometer (NanoDrop Technologies, Wilmington, DE).

### Library preparation and sequencing.

Sample libraries were prepared for sequencing using a NuGen Ultralow System V2 library kit (NuGen Technologies Inc., San Carlos, CA, USA). Samples were run on four lanes of an Illumina HiSeq 2000 instrument (Illumina, Inc., San Diego, CA, USA) with eight samples per lane (2 by 125 bp) at the Genomic and Microarray Core at the University of Colorado—Denver (Aurora, CO, USA).

### Processing metagenomic sequence data.

Raw sequence data were trimmed and filtered using Trimmomatic to remove low-quality reads (28). The ILLUMINACLIP command was used to remove Illumina TruSeq adaptors. Each read's first and last three base pairs were removed. Then, starting at the 3′ end of the read, a four-nucleotide sliding window calculated the average Phred score, and, if the score was lower than 15, that window was removed until the average quality score rose above 15. Finally, any reads with less than 36 nucleotides and their mates were removed from the data set. Trimmed reads were aligned to the Bos taurus (genome assembly UMD_3.1) and the draft *Bos indicus* (29) genomes, and these sequences were filtered out of the sample using the Burrows-Wheeler aligner (BWA) using default settings (21). The removal of these genomes created a nonhost read sample for each of the 32 samples.

### Resistome analysis.

Nonhost reads were aligned to the MEGARes database (10) using BWA with the default setting plus the “-N” option, which allowed all hits with no more than maxDiff differences to be found. A custom-developed Java-based script was used to parse the resulting SAM file such that the gene fraction, defined as the proportion of nucleotides in a given reference gene that aligned to at least one read, was calculated for each AMR gene in each sample (12). Only AMR genes with gene fractions of >80% were considered present in a sample and included in further analyses; this method aimed to decrease the number of false-positive identifications (11, 30).

The number of reads that were classified as matching a published gene accession number in the MEGARes database was tabulated, along with the corresponding hits to the higher levels of group, mechanism, and class of AMR, according to the hierarchical structure of the database (10). Analysis at the gene level was not performed to avoid biasing diversity measures as a result of nonuniform gene naming nomenclature and sparseness of many gene counts (31). Across all samples, genes that Noyes et al. (11) considered important to human health were specifically searched for at the gene level prior to downstream analysis. Hits to 33 AMR genes that were identified in <3 samples were removed from the analysis. Richness and Shannon's diversity were calculated on a data set with sparse features intact that was normalized as described below. The remaining counts were normalized in a two-step process. First, counts were normalized using cumulative sum scaling (CSS) with a default percentile of 0.5 for normalization, which accounted for different sampling depths across samples (32). After this, the equation of Li et al. (33) was adopted to account for differences in sequence lengths of AMR genes and bacterial loads in samples. Reads were aligned to the full Greengenes database using BWA with default paired-end settings to identify 16S sequences in all nonhost read samples (34). Then, the relative abundance of each AMR gene was calculated as follows:

(1) $$\text{Relative abundance}=\overset{n}{\underset{i=1}{\sum }}\frac{N_{AMR\text{-}normalized\;hits}/L_{AMR\;reference\;sequence}}{N_{16S\;sequence}/L_{16S\;sequence}}$$

with *N*_AMR-normalized hits_ as the number of cumulative-sum-scale (CSS)-normalized hits to one specific AMR gene, *L*_AMR reference sequence_ as the sequence length of the corresponding AMR gene, *N*_16S sequence_ as the number of hits to 16S sequences, and *L*_16S sequence_ as the average length of the 16S sequences in the Greengenes database. This equation allowed the expression of AMR gene-normalized counts as a ratio to bacterial 16S gene-normalized counts. After normalization, counts were aggregated to the AMR drug class and resistance mechanism for statistical analysis.

### Statistical analysis.

Comparisons were made using CSS-normalized counts according to Li et al. for reads classified as hits to AMR gene accession numbers. To assess systematic changes in resistome composition between treatment groups and over time, nonmetric multidimensional scaling (NMDS) ordination was performed using the Hellinger transformation and Euclidean distances to avoid overweighting of rare antimicrobial resistance determinants (ARDs) using R (version 3.3.0) with the Vegan metaMDS function (35). Separation among groups of interest was tested using analysis of similarities (ANOSIM) (36). Treatment main effects and interactions were examined using the Proc Mixed procedure in SAS (version 9.4). Adjustments for multiple comparisons were made using a Bonferroni correction with an initial α of 0.05, leading to a corrected critical value of α of 0.01 (0.05/5) for comparison at the level of the class of resistance and an α of 0.00385 (0.05/13) for comparisons made at the level of the mechanism of resistance. The main effects of day, treatment with CTC, and treatment with CCFA, as well as their interactions, were analyzed at the levels of AMR class and mechanism. These main effects and interactions were also used in the analysis of richness (the number of unique features in a sample; e.g., number of classes of AMR genes) and Shannon's diversity (the number and proportion of unique features in a sample). The effects of experiment replication and pen were dropped from the models as they were not significant. Sequencing lane (i.e., categorization of which samples were sequenced together on the same lane) was controlled as a random effect, and LSMEANS/PDIFF was used to compare means when a main effect or interaction was significant in relation to the respective critical value. All analyses considered pen the experimental unit.

### Comparison to previous studies.

To investigate whether results from previous investigations of the samples used in this study (performed using culture and qPCR) could be replicated using shotgun metagenomic sequencing, two additional analyses were performed. First, using the BWA output SAM file, a pearl script (https://github.com/lh3/bwa/blob/master/xa2multi.pl) was used to evaluate reads that could be assigned to multiple gene accession numbers in the AMR database with equal probability (as designated in the optional XA:Z sam field) by appending them to a new SAM file, allowing a single read to be classified as multiple hits. This new classification (i.e., allowing a read to be counted more than once) was then analyzed using the same pipeline for normalization and comparison of AMR relative abundance at the class and mechanism hierarchical levels.

Another *post hoc* procedure was used to investigate whether sequencing reads could be identified that matched the PCR primers used in previous studies (7, 8). Because the primers (Table 3) were small (20 to 24 bp), BW alignment was unsuccessful even after the minimum seed length was changed to 10 bp. Instead, Tablet (version 1.15.09.01) was used to visually assess the primer region to investigate reads that could match the sequences of interest (37). Each of the 32 samples was individually inspected to identify reads matching the sequences for primers targeting *tet*(A), *tet*(B), *bla*_CMY2_, and *bla*_CTX-M_.

**TABLE 3:** Primers used for PCRs and identified in shotgun metagenomic samples^*a*^

Gene name	Primer name^*b*^	Sequence	GenBank accession no.
*bla*_CMY-2_	585F	5′-CAG ACG CGT CCT GCA ACC ATT AAA-3′	AB212086
	1038R	5′-TAC GTA GCT GCC AAA TCC ACC AGT-3′	
	675F	5′-AGG GAA GCC CGT ACA CGT T-3′	
	738R	5′-GCT GGA TTT CAC GCC ATA GG-3′	
*bla*_CTX-M-24_	CTX-M(F)	5′-ATGTGCAGYACCAGTAA-3′	AY143430
	CTX-M(R)	5′-CCGCTGCCGGTYTTATC-3′	
*tet*(A)	tet(A)(F)	5′-GCTACATCCTGCTTGCCTTC-3′	X61367
	tet(A)(R)	5′-CATAGATCGCCGTGAAGAGG-3′	
*tet*(B)	tet(B)(F)	5′-TTGGTTAGGGGCAAGTTTTG-3′	J01830
	tet(B)(R)	5′-GTAATGGGCCAATAACACCG-3′	

a Adapted from Kanwar et al. (8).

b F, forward; R, reverse.

### Accession number(s).

Reads for all 32 samples described in this project have been deposited in the NCBI database under BioProject number PRJNA419351.

## Supplementary Material

Supplemental material
