# Supplementary material for: Effects of Ceftiofur and Chlortetracycline on the Resistomes of Feedlot Cattle
Source: Appl Environ Microbiol. 2018 Jun 18;84(13):e00610-18. doi: 10.1128/AEM.00610-18 (PMC6007121; doi:10.1128/AEM.00610-18)
Supplement: Supplemental material [file supp_84_13_e00610-18__index.html]

Supplemental material 

# Effects of Ceftiofur and Chlortetracycline on the Resistomes of Feedlot Cattle

## Supplemental material

- Supplemental file 1 -

  Classes, mechanism, and groups of antibiotic resistance genes found within the bovine fecal shotgun metagenomic samples (Table S1).

  XLSX, 38K
- Supplemental file 2 -

  Rarefaction curves of all samples at the species and antimicrobial resistance gene levels (Fig. S1); Tablet (v. 15.09.01) screenshot demonstrating the visual alignment of shotgun metagenomic reads with antibiotic resistance gene reference sequences (Fig. S2).

  PDF, 2.1M
