# Supplementary material for: Effects of Ceftiofur and Chlortetracycline on the Resistomes of Feedlot Cattle
Source: Appl Environ Microbiol. 2018 Jun 18;84(13):e00610-18. doi: 10.1128/AEM.00610-18 (PMC6007121; doi:10.1128/AEM.00610-18)
Supplement: Supplemental material [file AEM.00610-18_zam013188593s2.pdf]

Supplementary Material:

Effects of Ceftiofur and Chlortetracycline on the Resistome of Feedlot Cattle

Running Head: Effect of antimicrobial drugs on cattle resistome

Margaret D. Weinroth[a], H. Morgan Scott[b], Bo Norby[c], Guy H. Loneragan[d], Noelle R. Noyes[e], Pablo Rovira[f], Enrique Doster[e], Xiang Yang[f], Dale R. Woerner[a], Paul S. Morley[†e], Keith E. Belk[a†*]

Department of Animal Sciences, Colorado State University, Fort Collins, Colorado, USA[a]; Department of Veterinary Pathobiology, Texas A&M University, College Station, Texas, USA[b]; Department of Large Animal Clinical Sciences, Michigan State University, East Lansing, Michigan, USA[c]; Department of Animal & Food Sciences, Texas Tech University, Lubbock, Texas, USA[d]; Department of Clinical Sciences, Colorado State University, Fort Collins, Colorado, USA[e]; Instituto Nacional de Investigacion Agropecuaria, Treinta y Tres, Uruguay[f], Department of Animal Sciences, University of California, Davis, California, USA[f]

† P. S. M. and K. E. B. contributed equally to this work.

*Address correspondence to Keith Belk | keith.belk@colostate.edu

Figure S1: Rarefaction curve of all samples at the (A) species level and (B) antimicrobial resistance (AMR) genes level. The leveling off of the curve illustrates that an appropriate sampling depth was reached for the bacterial and AMR gene diversity of the community sampled.

Table S1: Classes, mechanism, and groups of antibiotic resistance genes found within the 32 bovine fecal shotgun metagenomic samples. *Included as an excel document.*

Figure S2: Screenshot of Tablet (v. 15.09.01; Milne *et al*., 2009) demonstrating the visual alignment of shotgun metagenomic reads to resistant gene database. Here, the alignment of shotgun samples to *tet*(A) is shown. Box 1 is the overall gene view showing shotgun hits to the entire length of the gene with the box with the shaded region denoting where on the gene you are looking. Box 2 is the AMR gene reference nucleotide sequence. Box 3 is the metagenomic hits that have aligned to the reference sequences. Box 4 is the defined primer region (5′ - GCTACATCCTGCTTGCCTTC- 3′).

Figure S1: Rarefaction Curve to Assess Sequencing Depth

Methods. Non-host reads were assigned taxonomic labels by Kraken version 0.10.6 beta (Wood and Salzberg, 2014), kraken-filter was used at a threshold of 0.20. Antimicrobial resistance gene counts were generated from the MEGARes pipeline as described in the main text methods. Rarefaction curves were constructed with the 'rarecurve' function from the Vegan package version 2.4-4.

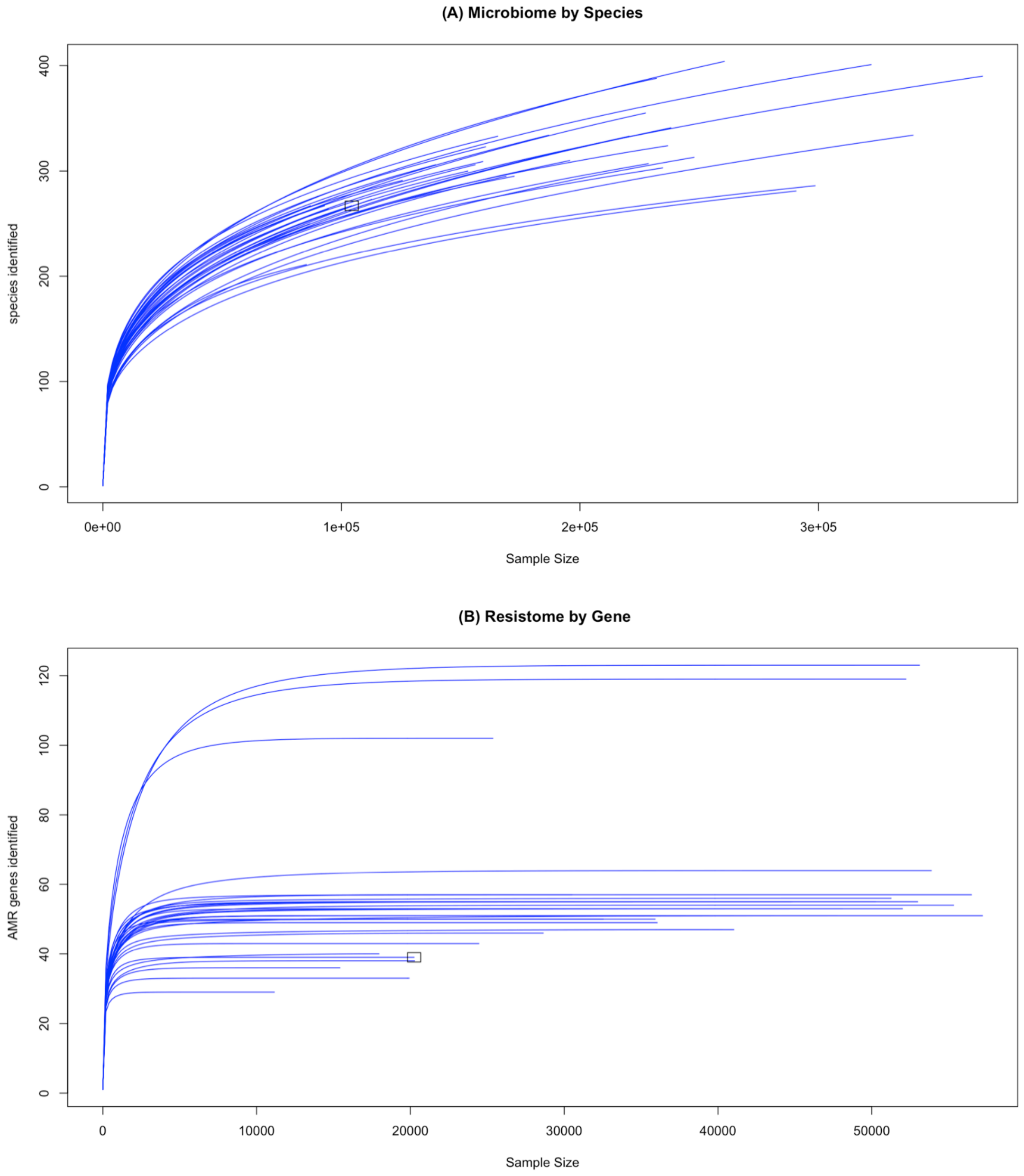

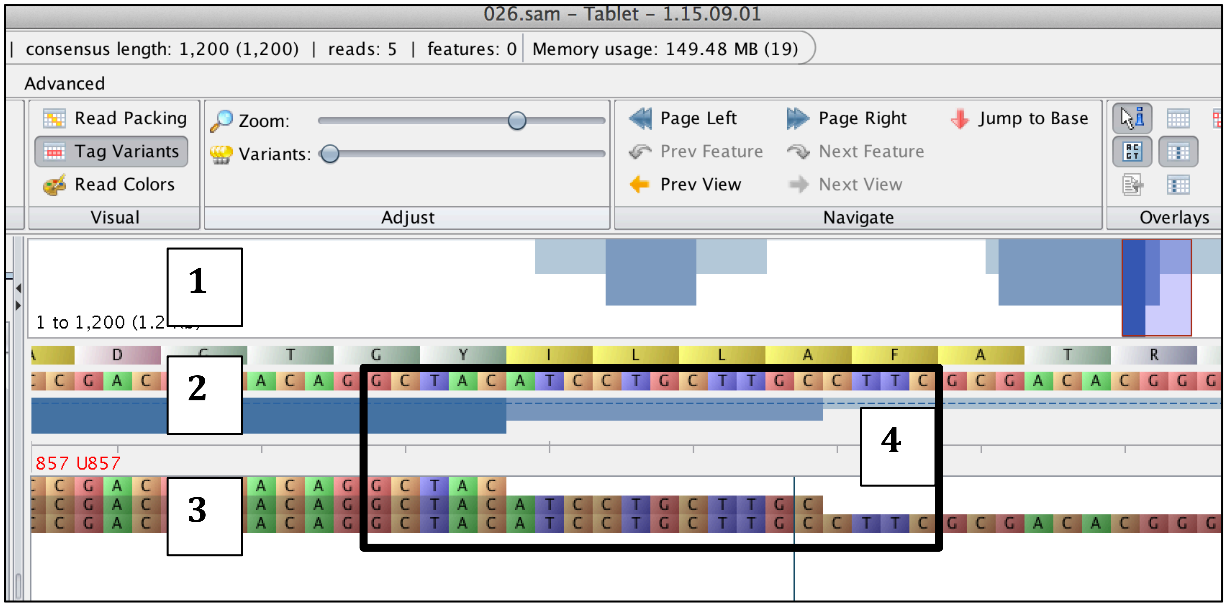

Figure S2: Screenshot of Tablet (v. 15.09.01; Milne *et al*., 2009) demonstrating the visual alignment of shotgun metagenomic reads to resistant gene database. Here, the alignment of shotgun samples to *tet*(A) is shown. Box 1 is the overall gene view showing shotgun hits to the entire length of the gene with the box with the shaded region denoting where on the gene you are looking. Box 2 is the AMR gene reference nucleotide sequence. Box 3 is the metagenomic hits that have aligned to the reference sequences. Box 4 is the defined primer region (5′ - GCTACATCCTGCTTGCCTTC- 3′).

Supplemental References

1. Wood DE, Salzberg SL. 2014. Kraken: ultrafast metagenomic sequence classification using exact alignments. Genome Biol 15:R46.

2. Milne I, Bayer M, Cardle L, Shaw P, Stephen G, Wright F, Marshall D. 2010. Tablet—next generation sequence assembly visualization. Bioinforma Oxf Engl 26:401–402.
